# New Appliances and Things Medical

**Published:** 1907-08-17

**Authors:** 


### NEW APPLIANCES AND THINGS MEDICAL.

[We shall be glad to receive, at our Office, 28 & 29 Southampton Street, Strand, London, W.C., from the manufacturers, specimens of all new preparations and appliances which may be brought out from time to time.]

### LYXHAYR.

(Lyxhayr, Ltd., Grove Mills, Mitcham, Surrey.)

In our issue of June 1, page 228, we gave a short notice of this vegetable fibre. We have now received a report from the large hospital where it has been thoroughly tested, and have satisfaction in publishing the following practical conclusions arrived at as to the merit of this article. The hospital secretary in question reports as follows :—"In accordance with your request I have given 'Lyxhayr' a trial of a little over two months. We had a mattress made of it, and put under a patient who was constantly in bed, and a day or two ago we had it opened and examined. The material showed no signs of matting or becoming hard, and when carded it did not break up or show any signs of deterioration. On the whole, it seems a suitable material for making hospital mattresses, and matron tells me that from a nursing point of view it has given every satisfaction." As the cost is less than a third of horse-hair, Lyxhayr is likely to come into general use, for it has the further advantage that 22 lbs. of Lyxhayr is found in practice to be the equivalent of 28 lbs. of horse-hair in a given mattress. This report is definite and convincing. Lyxhayr is worthy of being introduced into every economically managed hospital, and the experiment would seem to indicate that a longer test of the material in daily use is calculated to give every satisfaction. A substitute for horse-hair for mattress purposes has long been needed, and Lyxhayr promises to supply a fixed want in hospital wards.

